# Correlations between serum kidney injury molecule-1, cystatin C and immunosuppressants: A cross-sectional study of renal transplant patients in Bahrain

**DOI:** 10.7555/JBR.37.20220211

**Published:** 2024-03-26

**Authors:** Kannan Sridharan, Shamik Shah, Mona Al Hammad, Fatima Ali Mohammed, Sindhan Veeramuthu, Mona Abdulla Taher, Mustafa Mohamed Hammad, Lamees Jawad, Eman Farid

**Affiliations:** 1 Department of Pharmacology and Therapeutics, College of Medicine and Medical Sciences, Arabian Gulf University, Manama, Kingdom of Bahrain; 2 Department of Nephrology, Salmaniya Medical Complex, Manama, Kingdom of Bahrain; 3 Department of Internal Medicine, College of Medicine and Medical Sciences, Arabian Gulf University, Manama, Kingdom of Bahrain; 4 Salmaniya Medical Complex, Manama, Kingdom of Bahrain; 5 Department of Laboratory Medicine, Salmaniya Medical Complex, Manama, Kingdom of Bahrain; 6 Department of Microbiology, Immunology, and Infectious Diseases, College of Medicine and Medical Sciences, Arabian Gulf University, Manama, Kingdom of Bahrain

**Keywords:** KIM-1, cystatin C, mycophenolate mofetil, tacrolimus, everolimus, sirolimus, cyclosporine

## Abstract

Renal transplant patients receive several immunosuppressive drug regimens that are potentially nephrotoxic for treatment. Serum creatinine is the standard for monitoring kidney function; however, cystatin C (Cys C) and kidney injury molecule-1 (KIM-1) have been found to indicate kidney injury earlier than serum creatinine and provide a better reflection of kidney function. Here, we assessed Cys C and KIM-1 serum levels in renal transplant patients receiving mycophenolate mofetil, tacrolimus, sirolimus, everolimus, or cyclosporine to evaluate kidney function. We used both the Chronic Kidney Disease Epidemiology Collaboration (CKD-EPI) 2021 equation, which is based on creatinine and combined creatinine with Cys C, and the CKD-EPI 2012 equation, which is based on Cys C alone, to estimate glomerular filtration rate (GFR). Then, we assessed the association between serum KIM-1 and GFR < 90 mL per minute per 1.73 m^2^. We observed significantly higher serum Cys C levels in patients with the elevated serum creatinine, compared with those with normal serum creatinine. The estimated GFRs based on creatinine were significantly higher than those based on the other equations, while a significant positive correlation was observed among all equations. Serum KIM-1 levels were negatively correlated with the estimated GFRs by the CKD-EPI Cys C and the combined creatinine with Cys C equations. A serum KIM-1 level above 0.71 ng/mL is likely to indicate GFR < 90 mL per minute per 1.73 m^2^. We observed a significant correlation between serum creatinine and Cys C in our renal transplant patients. Therefore, serum KIM-1 may be used to monitor renal function when using potentially nephrotoxic drugs in renal transplants.

## Introduction

Kidney transplantation is the most common treatment for chronic kidney diseases and has a one-year graft survival rate of greater than 90%, with a rejection rate occurring in less than 15%^[1]^. One strategy proposed to improve the success rate of renal transplantation involves the incorporation of novel renal biomarkers for monitoring, such as kidney injury molecule-1 (KIM-1) and cystatin C (Cys C) in the evaluation of renal function^[2]^. KIM-1 protein, also known as T-cell immunoglobulin mucin receptor 1, is involved in viral infections, autoimmunity, immune tolerance, and atopic conditions^[3]^. KIM-1 has been found to be an early marker not only for acute kidney injury but also for predicting long-term renal outcomes^[4]^. Cys C is a 13 kDa protein produced by all nucleated cells and is exclusively filtered across the glomerulus, making it indicative of glomerular filtration rate (GFR)^[5]^.

Estimating the GFR has conventionally been carried out using serum creatinine alone that is known to be influenced by factors, such as muscle mass^[6]^. Recently, Cys C-based estimates for the GFR have been shown to predict the function of transplanted kidneys better than creatinine-based equations in Koreans^[7]^. A few studies have reported that serum Cys C is a more sensitive biomarker, capable of identifying damage earlier than creatinine in kidney transplant patients^[8–9]^. Both KIM-1 and Cys C have been shown to play a role in identifying rejection episodes in patients with renal transplantation^[10]^. Cys C-based GFR assessment is also thought to be more reliable both alone and in combination with serum creatinine and can be used to predict the risk of decompensation^[11]^. Hence, we hypothesized that serum Cys C and KIM-1 might be better predictors of renal function than serum creatinine in renal transplants, particularly on potential nephrotoxic drugs.

Mycophenolate mofetil, tacrolimus, sirolimus, everolimus, and cyclosporine are the most commonly used immunosuppressants for renal transplant patients^[12]^. The calcineurin inhibitors (cyclosporine and tacrolimus) and mammalian target of rapamycin (mTOR) inhibitors (sirolimus and everolimus) have been shown to cause arteriolopathy, striped interstitial fibrosis, glomerular congestion, and tubular microcalcification resulting in nephrotoxicity^[13]^. Clinical trials have reported differences in graft functions, graft survival, and GFR among immunosuppressive drugs^[14–15]^. Additionally, in clinical practice, combinations of immunosuppressive drugs are used, one of which is tacrolimus/sirolimus, and both drugs are reported to be nephrotoxic with poor long-term outcomes^[16]^. Thus, renal transplants are likely to receive drugs that are potentially nephrotoxic, leading to a deterioration in kidney function.

Patients receiving kidneys from the same donor and treated with tacrolimus showed a better creatinine clearance (87.7 [± 27.1] mL/min) than those treated with cyclosporin (60.3 [± 25.9] mL/min)^[17]^. Tacrolimus, cyclosporine, sirolimus, and everolimus have been attributed to varied incidences of nephrotoxicity^[18]^. Monitoring KIM-1 and Cys C in kidney transplant patients receiving immunosuppressive drugs can assist physicians in evaluating appropriate drugs and doses as well as in determining any decline in renal function much earlier than serum creatinine. However, because of cost concerns, they are not implemented in clinical practice^[19–20]^, and monitoring with serum creatinine has been the standard of care in almost all institutions.

Here, we carried out a descriptive study of serum KIM-1 and Cys C in kidney transplant patients and compared concentrations between different immunosuppressant regimens. Additionally, we have also estimated GFR based on Cys C, compared with the conventional approach using serum creatinine.

## Subjects and methods

### Study ethics and design

This cross-sectional study was carried out between June and September 2021 in the Nephrology Department, Salmaniya Medical Complex, Bahrain, after obtaining the approval from the Institutional Review Board (Approval No. E010-PI-6/21 and Serial No. 79080621) and informed consent from the recruited patients. We adhered to the latest Declaration of Helsinki guidelines.

### Study procedure

Patients who had undergone renal transplantation were recruited, if they were receiving any of the following immunosuppressive drugs: mycophenolate mofetil, tacrolimus, sirolimus, everolimus, or cyclosporine. Demographics, duration of kidney transplantation, current immunosuppressant regimen details, and serum creatinine were obtained. Blood samples were taken to estimate the concentrations of KIM-1 and Cys C. We estimated GFR using both the Chronic Kidney Disease Epidemiology Collaboration (CKD-EPI) 2021 equation, which is based on creatinine and combined creatinine with Cys C, and CKD-EPI 2012 equation that is based on Cys C^[21–22]^.

### Measures of KIM-1, Cys C, creatinine, and immunosuppressant levels

After collection, serum samples were centrifuged at 1500 *g* for 10 min, and the supernatant was stored in 0.5 mL aliquots at −80 ℃, pending analysis. Serum KIM-1 and Cys C were measured using the solid phase enzyme-linked immunosorbent assay (ELISA) kit obtained from Quantikine (R&D Systems, Minneapolis, MN, USA). Estimations of these biomarkers are based on the principles of the quantitative sandwich enzyme immunoassay technique, where a monoclonal antibody specific for the respective biomarkers is precoated on the microplate, on which standards and samples are pipetted. Cys C and KIM-1 were bound to the immobilized antibody that, after washing, were quantified. The co-efficient of variation ranged between 3.1% and 7% according to the manufacturer's instructions. Serum creatinine was measured using a modified kinetic Jaffe reaction method (Siemens, Newark, NJ, USA)^[23]^. Both Cys C and creatinine assays were traceable as recommended in the respective catalogs.

The trough therapeutic ranges were adhered to each immunosuppressive drug in the present study: tacrolimus (5–20 ng/mL), cyclosporine (90–150 ng/mL), and everolimus and sirolimus (4–12 ng/mL) using the ARCHITECT 1000i immunoassay analyzer (Abbott, Chicago, IL, USA). Serum levels within the abovementioned ranges were considered therapeutic, above the upper limits were considered supra-therapeutic, and below the lower limits were considered sub-therapeutic. The abovementioned immunosuppressants were measured using the chemiluminescent microparticle immunoassay technique with mouse monoclonal coated anti-drug antibodies in 3-(N-morpholino) propanesulfonic acid buffer with bovine serum albumin stabilizer.

### Statistical analysis

Descriptive statistics were used for demographic variables. The numerical variables are presented as median (range). Correlation analysis was performed by Pearson's correlation test. Comparison between two groups was performed by the Mann-Whitney *U* test, and the comparison among three independent groups was performed by the Kruskal-Wallis *H* test with Bonferroni's multiplicity correction. Receiver operating characteristics (ROC) curves were plotted to analyze the diagnostic ability of serum KIM-1 in predicting probabilities using GFR < 90 mL per minute per 1.73 m^2^. The Youden index was used to identify the threshold for serum KIM-1. A *P*-value < 0.05 was considered statistically significant, and 95% confidence intervals (CIs) were used for effect estimates. SPSS (version 27.0) was used for all statistical analyses.

## Results

### Demographics of the study participants

One-hundred and ninety-six participants were recruited, and descriptive demographics are shown in ***Table 1***. The majority of the participants were males and had systemic hypertension as a co-morbid disease.

**Table 1:** Demographic characteristics of the study participants

Variables	Values
Age (years, median [range])	49 (21–74)
Sex (male/female [*n*])	135/61
Duration of kidney transplantation (years, median [range])	6 (1–31)
Concomitant diagnoses (*n*)	
Systemic hypertension	159
Diabetes mellitus	80
Dyslipidemia	88
Hypothyroidism	9
Hyperuricemia	20

### Immunosuppressive drugs among the study participants

**Figure 1:**
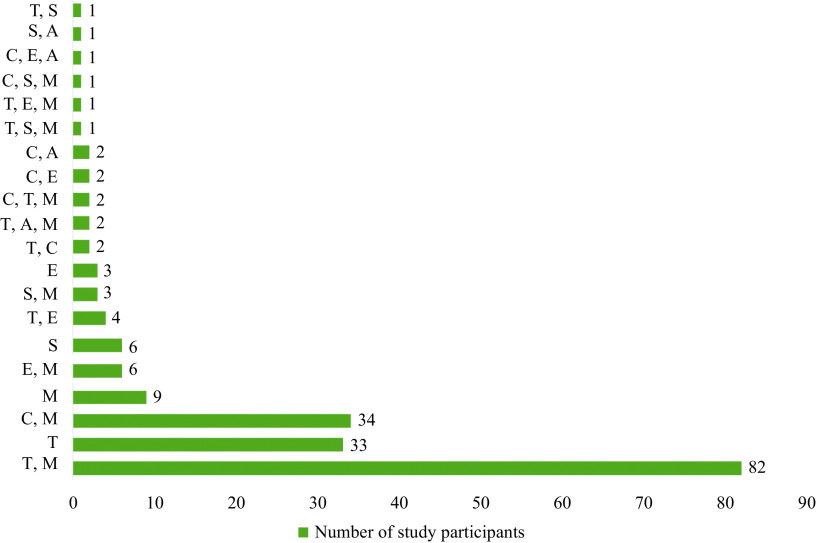
Immunosuppressive drug/s among the study participants.

The numbers of study participants receiving various immunosuppressive drug(s) are shown in ***Fig. 1***. The majority of participants, *i.e.*, 41.8% (*n* = 82), received tacrolimus and mycophenolate mofetil, followed by other drugs. The median (range) trough concentrations of tacrolimus, everolimus, and sirolimus were 5.9 (0.2–18.3) ng/mL, 5.6 (2.8–14.9) ng/mL, and 5.1 (3.7–16.1) ng/mL, respectively. Among the participants who received tacrolimus, 30.5% (*n* = 39) had sub-therapeutic trough levels, compared with 43.2% (*n* = 19) with cyclosporine, 33.3% (*n* = 2) with sirolimus, and 13.3% (*n* = 2) with everolimus. Meanwhile, 3.9% (*n* = 5) of the participants who received cyclosporine had a supra-therapeutic trough level. The majority received tacrolimus and mycophenolate, and a significant proportion of patients had sub-therapeutic trough concentrations of immunosuppressant drugs.

### Serum KIM-1 and Cys C among the study participants

**Figure 2:**
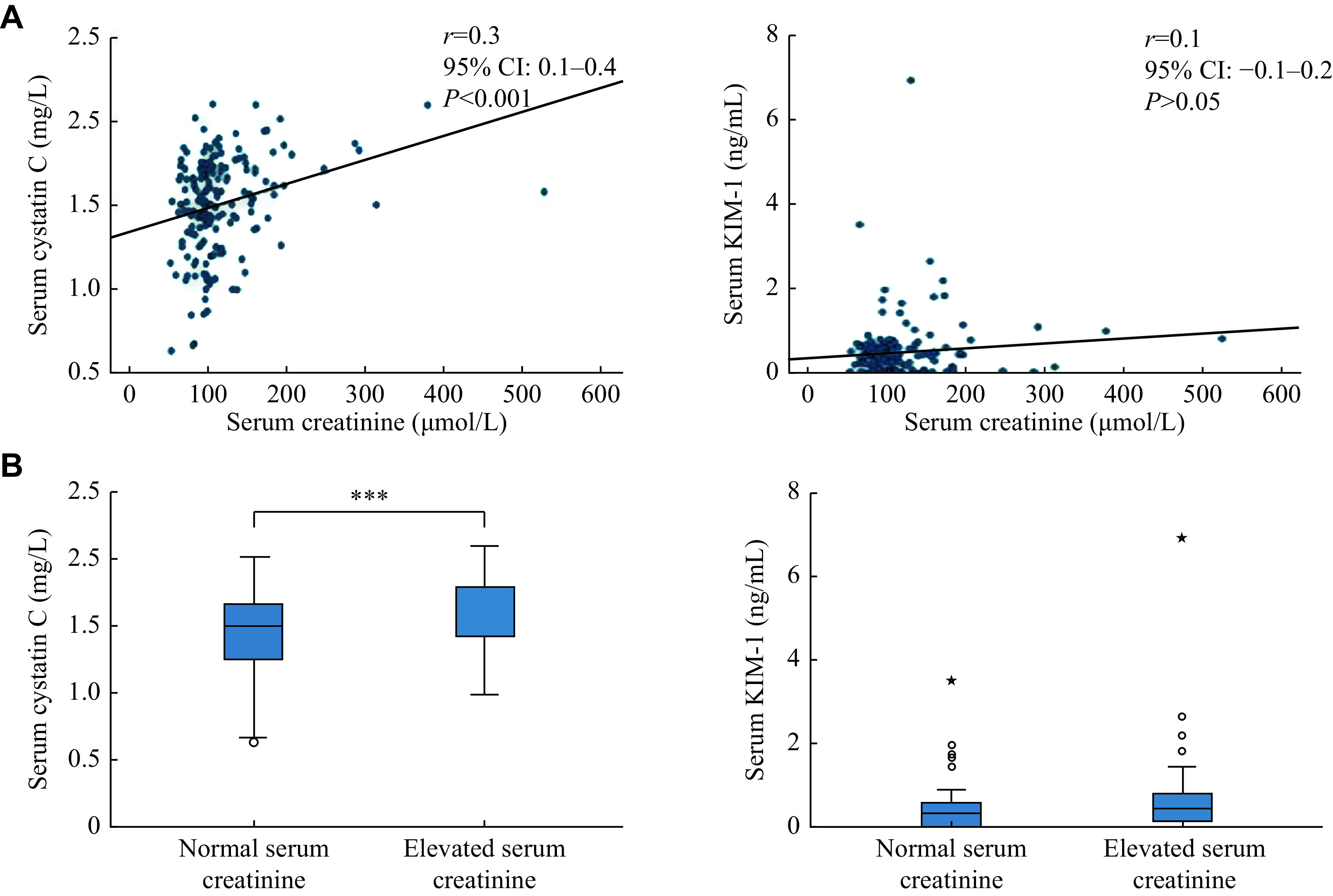
Correlation of serum creatinine with KIM-1 and Cys C.

The serum levels (median [range]) of KIM-1 and Cys C among the study participants were 0.4 (0–6.9) ng/mL and 1.5 (1.2–2.1) mg/L, respectively. A significant correlation was observed between serum levels of creatinine and Cys C (correlation coefficient [*r*] = 0.3; 95% confidence interval [CI]: 0.1–0.4; *P* < 0.001), but not between creatinine and KIM-1 (*r* = 0.1, 95% CI: −0.1–0.2; *P* > 0.05) (***Fig. 2A***). A significantly higher Cys C concentration was observed in patients with elevated serum creatinine (*P* < 0.001), compared with those with normal serum creatinine, while KIM-1 concentrations were similar between the two groups (***Fig. 2B***). No significant correlations were observed of the duration of kidney transplantation with serum Cys C (*P* > 0.05) and KIM-1 (*P* > 0.05) concentrations.

### Correlation between serum levels of KIM-1, Cys C, and creatinine and immunosuppressive drugs

**Figure 3:**
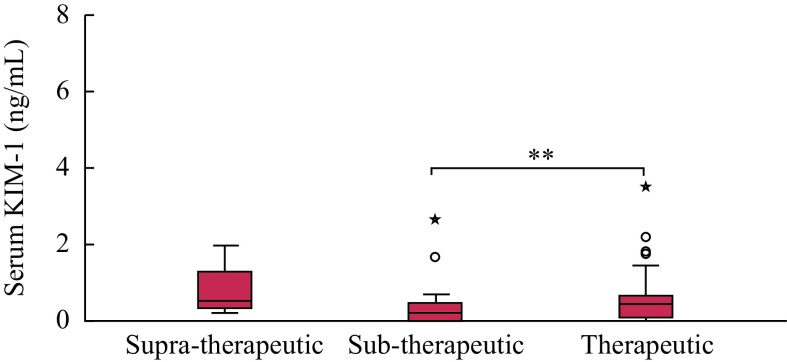
Serum KIM-1 concentrations among different categories of immunosuppressive drug levels.

No significant differences were observed in serum levels of KIM-1, Cys C, and creatinine among the patients who received various immunosuppressive drug/s (***Supplementary Fig. 1***, available online). No significant (*P* > 0.05) differences in serum Cys C levels (median [range]) were observed among those with therapeutic (1.5 [0.8–2.1] mg/L), sub-therapeutic (1.4 [1.2–2.1] mg/L), and supra-therapeutic (1.4 [1.1–1.7] mg/L) trough levels of immunosuppressive drugs. However, the participants with sub-therapeutic trough levels of immunosuppressants had significantly (*P* < 0.05) lower serum levels of KIM-1, compared with those with therapeutic trough drug levels (***Fig. 3***). No significant (*P* > 0.05) differences were observed in serum levels (median [range]) of creatinine among those with therapeutic (94.5 [44.0–531.0] µmol/L), supra-therapeutic (90.0 [84.0–97.0] µmol/L), and sub-therapeutic (100.5 [44.0–312.0] µmol/L) trough drug levels.

### The estimated GFRs based on CKD-EPI equations

**Figure 4:**
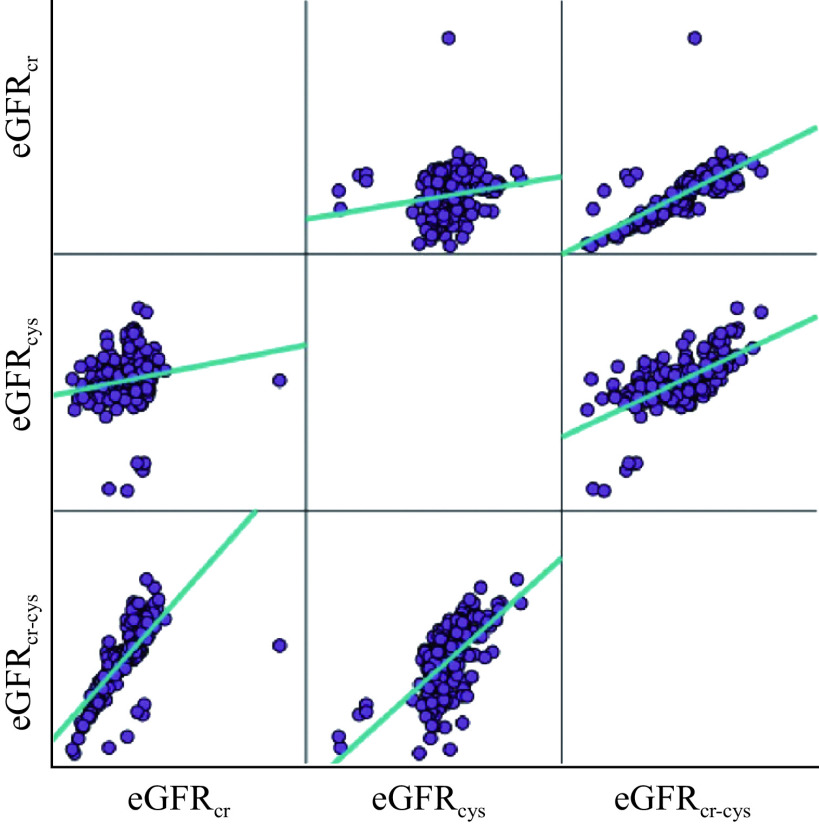
Correlation analyses among the eGFRs based on creatinine, Cys C, and combined creatinine with Cys C.

The median (range) GFRs estimated by the CKD-EPI creatinine equation (79.5 [11.0–282.0] mL per minute per 1.73 m^2^) was significantly (*P* < 0.001) greater than those by the CKD-EPI Cys C equation (45.0 [27.0–121.0] mL per minute per 1.73 m^2^) or the CKD-EPI combined creatinine with Cys C (58.0 [20.0–117.0] mL per minute per 1.73 m^2^). However, we observed significantly positive correlations of the GFRs estimated by the CKD-EPI equations between creatinine and Cys C (*r* = 0.2, 95% CI: 0.1–0.3, *P* < 0.05), and between Cys C and combined creatinine with Cys C (*r* = 0.7, 95% CI: 0.6–0.7, *P* < 0.001), as well as between creatinine and combined creatinine with Cys C (*r* = 0.7, 95% CI: 0.6–0.8, *P* < 0.001) (***Fig. 4***).

**Figure 5:**
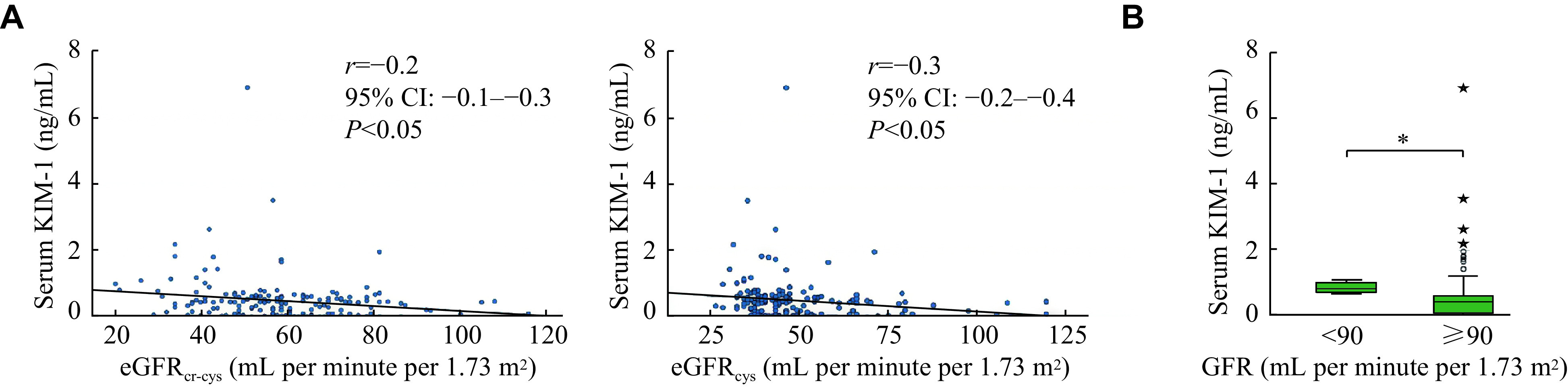
Serum levels of KIM-1 and eGFRs were estimated based on Cys C and combined creatinine with Cys C.

**Figure 6:**
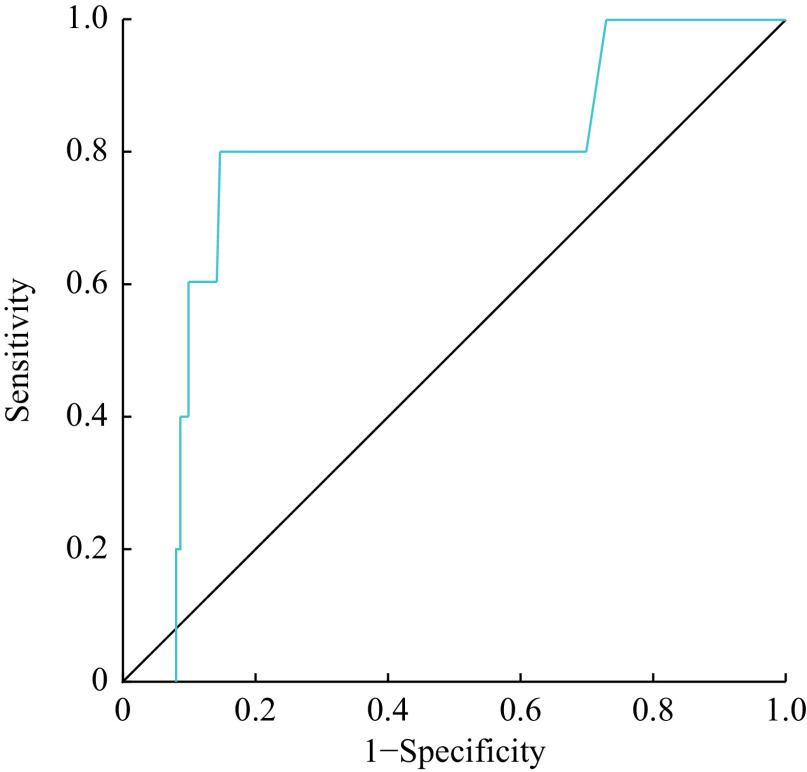
ROC curve for serum KIM-1 and GFR category.

No significant differences were observed in the estimated GFRs (eGFRs) by all the equations among the immunosuppressive drug/s (***Supplementary Table 1***, available online). Similarly, no significant differences were observed in the eGFRs among therapeutic, sub-therapeutic, and supra-therapeutic trough drug levels (***Supplementary Table 2***, available online). A significant negative correlation was observed between serum levels of KIM-1 and eGFRs estimated by the CKD-EPI combined creatinine with Cys C equation (*r* = −0.2, 95% CI: −0.1–−0.3, *P* < 0.05) and between serum levels of KIM-1 and eGFRs estimated by the CKD-EPI Cys C equation (*r* = −0.3, 95% CI: −0.2–−0.4, *P* < 0.05) (***Fig. 5A***). Serum levels of KIM-1 were significantly higher in participants with eGFR < 90 mL per minute per 1.73 m^2^, compared with those with eGFR ≥ 90 mL per minute per 1.73 m^2^(***Fig. 5B***). The ROC plot (***Fig. 6***) indicates 0.71 ng/mL as the optimal cut-off value for serum KIM-1 levels, above which the eGFR is likely to be below 90 mL per minute per 1.73 m^2^. Thus, serum KIM-1 levels were negatively correlated with eGFRs and also better-predicted individuals with significant reductions in eGFR.

## Discussion

The present study was designed to evaluate the serum concentrations of KIM-1 and Cys C in 196 renal transplant patients receiving immunosuppressive drugs. Tacrolimus and mycophenolate mofetil comprised the major immunosuppressive drugs received by the study participants. We observed a significant correlation between serum creatinine and Cys C, as well as significantly higher Cys C concentrations in patients with the elevated serum creatinine, compared with those within a normal range. Serum KIM-1 levels were significantly lower in patients with sub-therapeutic trough levels than in those with therapeutic trough drug levels of the drug. The estimated GFRs, based on the CKD-EPI creatinine equation, were significantly higher than other equations; yet, a significant correlation was observed overall among all equations. Serum KIM-1 levels were negatively correlated with GFR estimated using CKD-EPI combined creatinine and Cys C-based equations.

Maintenance of immunosuppressive drug regimens used in renal transplant patients varies widely between transplant units, despite no clear identification of superior benefits with one regimen over another^[24]^. However, considering the immunological risks associated with graft rejection, opportunistic infections, and secondary malignancies, recent guidelines have recommended a calcineurin inhibitor, preferably tacrolimus, with an anti-proliferative drug, ideally mycophenolate mofetil^[25]^, which is in accordance with the observation in the present study. We did not observe any significant differences in serum levels of creatinine, KIM-1, Cys C, and estimated GFRs by all the equations among the immunosuppressive drug/s. Calcineurin inhibitors, such as tacrolimus, have been found with nephrotoxicity and consequently graft failure^[26]^. Also, recent evidence favored alloimmunity over calcineurin used in such instances^[27]^. We did not observe any significant differences in serum biomarkers or GFRs between those receiving calcineurin inhibitors and those who did not. However, these should be interpreted with caution, because we did not assess serial changes in the biomarkers or GFRs, but rather these results were of a point-estimate.

Despite a significant correlation being observed between Cys C and serum creatinine, the strength of the correlation was weak. A modest correlation has been reported between serum creatinine and Cys C^[28]^. Differences in the estimation technique of serum creatinine have also been observed to influence this correlation^[29]^. One of the plausible reasons for the weaker correlation observed in the present study may be because of the differences in estimation methods (the modified Jaffe method). Secondly, serum creatinine levels are influenced by body weight, and renal transplant patients, because of reasons such as dietary restrictions, poor appetite, and polypharmacy, may have significantly altered creatine levels that do not provide an accurate estimation of renal dysfunction, which is unlike Cys C. Although we observed good correlations among the three CKD-EPI equations, the combined creatinine and Cys C-based equation had stronger correlations with creatinine and Cys C. The combined Cys C and creatinine equation showed more accuracy than either alone, as reported in a previous study^[30]^.

We observed a lower GFR estimated from Cys C and the combined creatinine and Cys C-based equations, compared with creatinine alone, which corresponds with findings from a recent study^[6]^. The inter-assay differences in the analytical methods for serum creatinine and Cys C could also possibly explain variations, because a gold standard remains unavailable for Cys C^[31]^. Until recently, CKD-EPI equations have been adjusted for ethnicity. However, recent studies have suggested that CKD-EPI equations perform better without intercalating ethnicity weights^[19,32]^. Here, we used the latest CKD-EPI 2021 equations without considering ethnicity. The lack of anthropometric data for the CKD-EPI equations offers distinct advantages not only in adult populations but also in children as observed in a recent study, where an optimal strategy for estimating GFR was observed to be the combination of creatinine-based on age and Cys C methods^[33]^.

Serum KIM-1 levels were negatively correlated with GFR in the present study, which substantiates findings from previous studies, despite using different GFR measures^[34]^. KIM-1 levels elevated because of renal injury, and even successful kidney transplant patients were observed with kidney injury^[35]^, as in the present study, which is also substantiated by the significantly elevated KIM-1 levels in patients with eGFR < 90 mL per minute per 1.73 m^2^. Significantly lower serum KIM-1 levels were observed in those with subtherapeutic trough concentrations of the immunosuppressive drugs, but no significant differences in eGFR values were observed in this sub-population. It is plausible to observe sub-clinical renal insults with therapeutic concentrations of immunosuppressive drugs, which can possibly explain the low serum KIM-1 concentrations.

The strength of the present study was a relatively larger number of study participants, compared with previous studies^[36]^. Moreover, the GFR was estimated in the present study using the latest CKD-EPI guidelines without considering the ethnic variation, and we also estimated the optimal cut-off for serum KIM-1 levels in renal transplant patients. However, the present study was limited by serial changes in the biomarkers over time. We also could not measure GFR using exogenous markers, and details regarding rejection episodes were not available for the study participants. Patients with CKD have been observed with skeletal muscle wasting/muscle atrophy^[37]^; thus, for this reason, it is biologically plausible that there were a few participants with higher eGFR values based on serum creatinine. Future studies shall consider assessing the absolute skeletal mass for delineating this association.

### Conclusion

There is a significant correlation between serum creatinine and Cys C in Bahraini renal transplant patients. The monitoring of serum KIM-1 may be used as a reliable predictor for GFR in renal transplant patients. A cut-off value of 0.71 ng/mL was established for serum KIM-1, above which patients are expected to have sub-optimal GFR.

## SUPPLEMENTARY DATA

Supplementary data to this article can be found online.
