# Supplementary material for: Correlations between serum kidney injury molecule-1, cystatin C and immunosuppressants: A cross-sectional study of renal transplant patients in Bahrain
Source: J Biomed Res. 2024 Mar 26;38(3):269–77. doi: 10.7555/JBR.37.20220211 (PMC11144937; doi:10.7555/JBR.37.20220211)
Supplement: Supplementary file 1 — Supplementary data to this article can be found online. [file jbr-38-3-269-S1.pdf]

Available online at www.jbr-pub.org.cn

Open Access at PubMed Central

Journal of Biomedical Research, 2024 0(000): 1–3

Supplementary Data

JBR

Correlations between serum kidney injury molecule-1, cystatin C and immunosuppressants: A cross-sectional study of renal transplant patients in Bahrain

Kannan Sridharan[1,✉], Shamik Shah[2,3], Mona Al Hammad[4], Fatima Ali Mohammed[2], Sindhan Veeramuthu[1], Mona Abdulla Taher[2], Mustafa Mohamed Hammad[4], Lamees Jawad[5], Eman Farid[5,6]

[1]*Department of Pharmacology and Therapeutics, College of Medicine and Medical Sciences, Arabian Gulf University, Manama, Kingdom of Bahrain;*
[2]*Department of Nephrology, Salmaniya Medical Complex, Manama, Kingdom of Bahrain;*
[3]*Department of Internal Medicine, College of Medicine and Medical Sciences, Arabian Gulf University, Manama, Kingdom of Bahrain;*
[4]*Salmaniya Medical Complex, Manama, Kingdom of Bahrain;*
[5]*Department of Laboratory Medicine, Salmaniya Medical Complex, Manama, Kingdom of Bahrain;*
[6]*Department of Microbiology, Immunology, and Infectious Diseases, College of Medicine and Medical Sciences, Arabian Gulf University, Manama, Kingdom of Bahrain.*

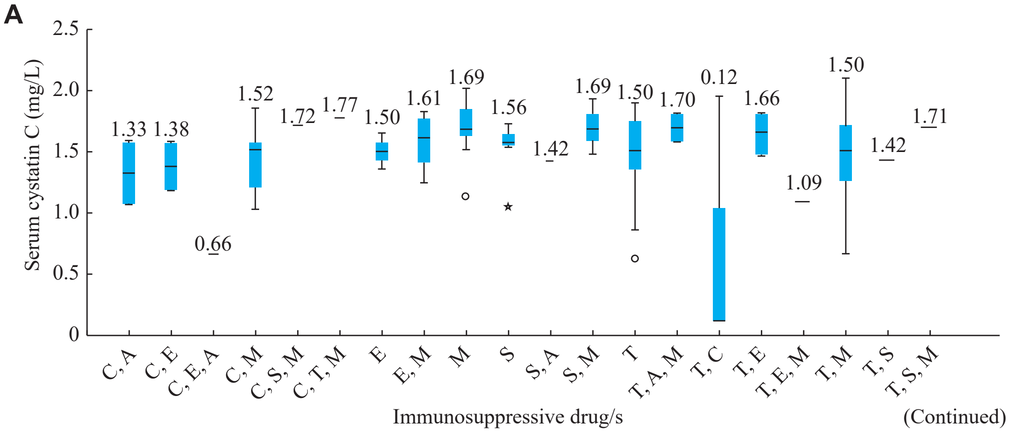

(Continued)

✉Corresponding author: Kannan Sridharan, Department of Pharmacology and Therapeutics College of Medicine and Medical Sciences, Arabian Gulf University, PO Box 26671, Road 2904, Manama, Kingdom of Bahrain. E-mail: skannandr@gmail.com.

Received: 25 September 2022; Revised: 24 October 2022; Accepted: 06 November 2022; Published online: 26 March 2024

CLC number: R699.2, Document code: A

The authors reported no conflict of interests.

This is an open access article under the Creative Commons Attribution (CC BY 4.0) license, which permits others to distribute, remix, adapt and build upon this work, for commercial use, provided the original work is properly cited.

© 2024 by Journal of Biomedical Research.

https://doi.org/10.7555/JBR.36.20220211

(Continued)

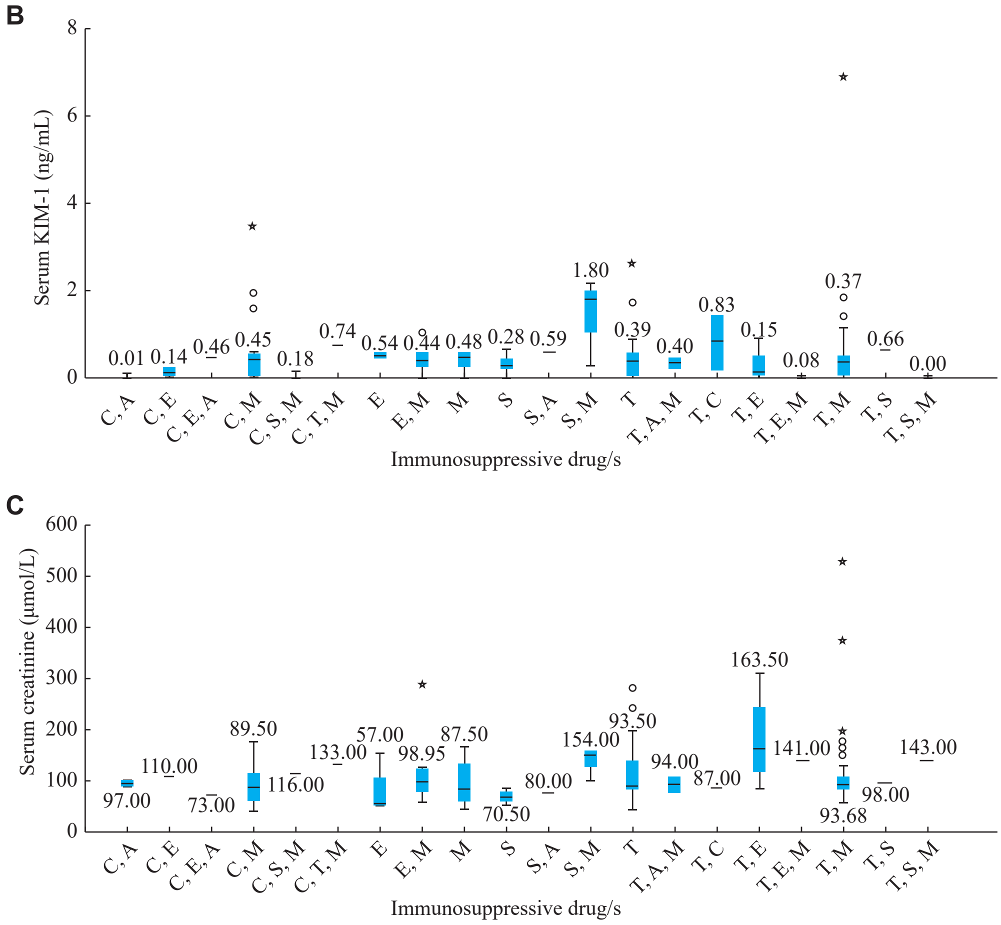

Supplementary Fig. 1 **Serum levels of Cys C, KIM-1, creatinine and immunosuppressive drug/s.** Serum levels of Cys C and KIM-1 were measured by the enzyme-linked immunosorbent assay, while creatinine was measured using the modified kinetic Jaffe reaction method. Data are presented as median and interquartile range. The number above each group indicates the median. Small white circles indicate outlier values lying 1.5 times the interquartile range below the first quartile; asterisks represent the values above 3 times below the first quartile. Comparisons of the serum levels of Cys C (A), KIM-1 (B), and creatinine (C) in the participants who received various immunosuppressive drug/s were performed by the Kruskal-Wallis H test. Abbreviations: Cys C, cystatin C; C, cyclosporine; A, azathioprine; E, everolimus; M, mycophenolate mofetil; T, tacrolimus; S, sirolimus.

Supplementary Table 1 **Comparison of eGFRs among participants received various immunosuppressive drug/s**

Drugs	Creatinine-based eGFR (mL per minute per 1.73 m^2)	Cys C-based eGFR (mL per minute per 1.73 m^2)	Creatinine and Cys C-based eGFR (mL per minute per 1.73 m^2)
C, A	81.5 (78.0–85.0)	59.5 (46.0–73.0)	69.0 (59.0–79.0)
C, E	67.0	44.0	55.0
C, E, A	96.0	122.0	109.0
C, M	93.0 (32.0–118.0)	45.5 (36.0–81.0)	59.0 (34.0–93.0)
C, M, S	64.0	40.0	49.0
C, M, T	45.0	38.0	39.0
E	100.0 (46.0–108.0)	44.0 (37.0–55.0)	58.0 (50.0–66.0)
E, M	59.5 (19.0–113.0)	42.0 (35.0–62.0)	48.5 (26.0–80.0)
M	76.5 (34.0–110.0)	35.0 (33.0–43.0)	48.5 (31.0–68.0)
S	101.5 (95.0–112.0)	44.0 (38.0–71.0)	67.0 (57.0–82.0)
S, A	118.0	56.0	79.0
S, M	47.0 (38.0–75.0)	42.0 (32.0–49.0)	43.0 (34.0–59.0)
T	79.5 (24.0–132.0)	44.0 (35.0–111.0)	57.0 (29.0–116.0)
T, A, M	87.0 (57.0–117.0)	42.5 (37.0–48.0)	57.5 (43.0–72.0)
T, C	69.0	31.0	44.0
T, E	40.0 (22.0–108.0)	44.5 (39.0–53.0)	40.0 (32.0–64.0)
T, E, M	36.0	65.0	48.0
T, M	82.0 (11.0–282.0)	46.0 (27.0–121.0)	59.5 (20.0–117.0)
T, S	53.0	45.0	48.0
T, S, M	58.0	44.0	49.0
Kruskal-Wallis H value; *P*-value	24.2; 0.3	19.2; 0.6	22.0; 0.4

The estimated glomerular filtration rates (eGFRs) were calculated by the Chronic Kidney Disease Epidemiology Collaboration (CKD-EPI) 2021 equation, which is based on creatinine and combined creatinine with Cys C, and the CKD-EPI 2012 equation that is based on Cys C alone. Data are presented as median (range). Groups with one or two participants are represented only with the median values. The Kruskal-Wallis *H* test was used for statistical comparisons.
Abbreviations: Cys C, cystatin C; C, cyclosporine; A, azathioprine; E, everolimus; M, mycophenolate mofetil; T, tacrolimus; S, sirolimus.

Supplementary Table 2 **Comparison of GFRs among categories of drug trough levels**

Drug trough levels	Creatinine-based GFR (mL per minute per 1.73 m^2)	Cys C-based GFR (mL per minute per 1.73 m^2)	Creatinine and Cys C-based GFR (mL per minute per 1.73 m^2)
Supra-therapeutic	94.0 (86.0–99.0)	66.0 (39.0–72.0)	73.0 (58.0–82.0)
Sub-therapeutic	73.5 (22.0–122.0)	47.5 (29.0–121.0)	59.0 (29.0–117.0)
Therapeutic	80.0 (11.0–282.0)	45.0 (27.0–110.0)	58.5 (20.0–106.0)
Kruskal-Wallis H value; *P*-value	2.4; 0.5	7.4; 0.1	2.6; 0.5

The estimated glomerular filtration rates (eGFRs) were calculated by the Chronic Kidney Disease Epidemiology Collaboration (CKD-EPI) 2021 equation, which is based on creatinine and combined creatinine with Cys C, and the CKD-EPI 2012 equation that is based on Cys C alone. The data are presented as median (range). The Kruskal-Wallis *H* test was used for statistical comparisons.
